# Key genes with prognostic values in suppression of osteosarcoma metastasis using comprehensive analysis

**DOI:** 10.1186/s12885-020-6542-z

**Published:** 2020-01-28

**Authors:** Mi Li, Xin Jin, Hao Li, Gang Wu, Shanshan Wang, Caihong Yang, Sisi Deng

**Affiliations:** 1 Department of Orthopedics, Tongji Hospital, Tongji Medical College, Huazhong University of Science and Technology, Wuhan, 430030 China; 2 Department of Digestive Surgical Oncology, Union Hospital, Tongji Medical College, Huazhong University of Science and Technology, Wuhan, 430022 China; 3 Cancer Center, Union Hospital, Tongji Medical College, Huazhong University of Science and Technology, Wuhan, 430022 China

**Keywords:** Osteosarcoma, Metastasis, Prognosis, Protein-protein interaction network, Differentially expressed genes

## Abstract

**Background:**

Osteosarcoma is a primary malignant tumor originating from mesenchymal tissue, with a poor distant metastasis prognosis. The molecular mechanisms of osteosarcoma metastasis are extremely complicated.

**Methods:**

A public data series (GSE21257) was used to identify differentially expressed genes (DEGs) in osteosarcoma patients that did, or did not, develop metastases. Functional enrichment analysis, a protein-protein interaction network, and survival analysis of DEGs were performed. DEGs with a prognostic value were considered as candidate genes and their functional predictions, different expression in normal and malignant tissues, and immune infiltration were analyzed.

**Results:**

The DEGs were mainly enriched in the immune response. Three candidate genes (ALOX5AP, CD74, and FCGR2A) were found, all of which were expressed at higher levels in lungs and lymph nodes than in matched cancer tissues and were probably expressed in the microenvironment.

**Conclusions:**

Candidate genes can help us understand the molecular mechanisms underlying osteosarcoma metastasis and provide targets for future research.

## Background

Osteosarcoma is a primary malignant tumor originating from mesenchymal tissue. The annual incidence is similar worldwide, ranging from 1 to 4 in 1 million. Although the overall incidence of osteosarcoma is not high, it is the most common type of bone and soft tissue tumors, accounting for 40.51% of primary malignant bone tumors. With improvements in limb salvage surgery and neoadjuvant chemotherapy, the 5-year survival rate of non-metastatic patients is about 65–70% [1]. Unfortunately, distant metastases are found in about 20% of patients, 90% of which are lung metastases [2]. Once distant metastasis occurs, the 5-year survival rate is only 15–30% [3–5]. However, the mechanisms of osteosarcoma metastasis are still largely unknown.

In recent years, bioinformatics has been widely used to reveal tumor progression and the internal mechanism of carcinogenesis at the genome level for many cancer types. In particular, there are many bioinformatics web tools that can help us analyze relevant data, with standardized and visual results. Although microarray data for osteosarcoma are still limited, some hidden and interesting information like the expression of key genes [6–8], microRNAs [9] and co-expression modules [10] in osteosarcoma and drug resistance in osteosarcoma patients [11] could be found.

In this study, a series of mRNA data was analyzed to obtain differentially expressed genes (DEGs) between osteosarcoma patients that did, or did not, develop metastases. Subsequently, a protein-protein interaction (PPI) network of the DEGs was constructed. Gene Ontology (GO), Kyoto Encyclopedia of Genes and Genomes (KEGG) pathway enrichment analyses, and survival analysis were used to identify candidate genes. Furthermore, we analyzed function predictions, different expression in normal and malignant human tissues, and immune infiltration analysis of the candidate genes to confirm their function and distribution. In conclusion, 24 DEGs and three candidate genes were identified.

## Methods

### Identification of DEGs and PPI network construction

A public series submitted by Buddingh et al. in 2011, GSE21257 [12], was downloaded from the Gene Expression Omnibus database (GEO, http://www.ncbi.nlm.nih.gov/geo, RRID: SCR_005012) [13]. The series contains 53 pre-chemotherapy biopsy samples from osteosarcoma patients that developed metastases (*n* = 34) and that did not develop metastases within 5 yrs. (*n* = 19). The biopsy tissue contained the tumor cells and microenvironment around the tumor. All the expression data were analyzed via the R language (version 3.5.1) BIOCONDUCTOR package, and the DEGs were screened using the LIMMA package at a statistical significance Benjamini and Hochberg false discovery rate-adjusted *p*-value cutoff of 0.05 and an absolute value of fold change greater than 2. The online Search Tool for the Retrieval of Interacting Genes (STRING, http://string-db.org, RRID: SCR_005223) [14] is a database of known and predicted protein-protein interactions. We used STRING to find observed co-expression of the DEGs in humans and constructed a PPI network of the DEGs with statistical significance of interaction scores > 0.4 (medium confidence score).

### GO and pathway enrichment

The GO and KEGG pathway enrichment analyses were performed using DAVID (https://david.ncifcrf.gov/, RRID: SCR_001881) [15]. The biological process (BP) analysis, cellular component (CC) analysis, molecular function (MF) analysis [16], and KEGG [17] pathway enrichment analysis of the DEGs were carried out and *p*-values < 0.05 were considered to indicate statistical significance. Moreover, a biological process analysis of the hub genes was constructed and visualized using the Biological Networks Gene Ontology tool (BiNGO, RRID: SCR_005736) [18] plugin of Cytoscape (version 3.6.1, RRID: SCR_003032) [19].

### Survival analysis of the DEGs

PROGgeneV2 (http://genomics.jefferson.edu/proggene) [20] is a tool that can be used with publicly available data to study the prognostic implications of genes. All the DEGs were input into the database separately and overall survival plots (Kaplan Meier, KM plots) were created based on the cohort divided at the median of the given gene expression. PROGgeneV2 uses the SURVIVAL package of R for the hypothesis test. The DEGs that had *p*-values < 0.05 were considered as candidate genes and were analyzed further.

### Function predictions of the candidate genes

GeneMANIA (http://www.genemania.org, RRID: SCR_005709) [21] is a flexible, user-friendly open-source tool. Besides constructing the PPI network, the web tool can display an interactive functional association network, illustrating the relationships among genes. The advanced statistical options used were max resultant genes = 20, max resultant attributes = 10, and the automatically selected network weighting method. These analyses were conducted using the *Homo sapiens* database.

### Different expression of candidate genes in normal and malignant human tissues

The SAGE Anatomic Viewer, part of the online Serial Analysis of Gene Expression database (SAGE, http://www.ncbi.nlm.nih.gov/SAGE, RRID: SCR_000796) [22], was used to display candidate gene expression in normal and malignant human tissues. The related expression levels were based on the analysis of counts of SAGE tags, ordered by color.

### Immune infiltration analysis of the candidate genes

Tumor IMmune Estimation Resource (TIMER, https://cistrome.shinyapps.io/timer/) [23] is a comprehensive web server for systematic analysis of immune infiltrates across diverse cancer types. When we input the candidate gene symbols for at least one cancer type, scatterplots were generated and displayed showing the purity-corrected partial Spearman’s correlations and statistical significance. Tumor purity is expected to have negative associations with high levels of expression in the microenvironment, while the opposite is true for the tumor cells. Unfortunately, there is no available data for osteosarcoma, so we chose SARC (sarcoma), OV (ovarian serous cystadenocarcinoma), LUSC (lung squamous cell carcinoma), LIHC (liver hepatocellular carcinoma), and BRCA (breast invasive carcinoma) as the multi-cancer types.

## Results

### Identification of DEGs and PPI network construction

**Fig. 1:**
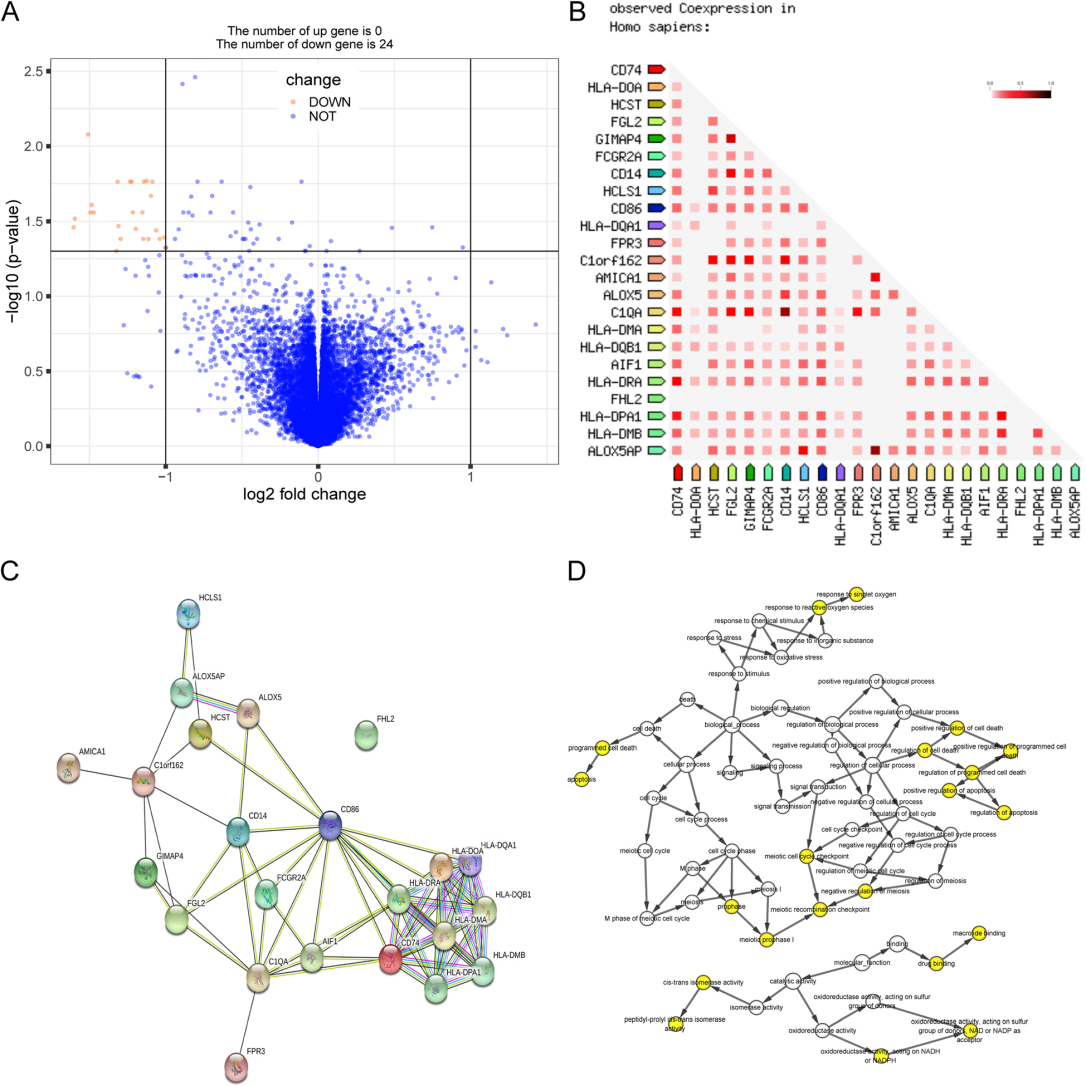
Volcano plot, observed co-expressed genes, protein-protein interaction (PPI) network, and biological process analysis of DEGs. The DEGs were screened with criteria of *p* < 0.01 and absolute value logFC (fold change) > 1; the red dots represent downregulated genes and the blue dots represent unchanged genes (**a**). The observed co-expressed genes of DEGs in *Homo sapiens* are shown in triangular matrices; the intensity of color indicates the level of confidence that two proteins are functionally associated (**b**). The PPI network of the DEGs; the network nodes represent proteins and the edges represent the protein-protein associations (**c**). Biological process analysis of the DEGs was performed and visualized using BiNGO; the color depth of the nodes refers to the corrected *p* values of the ontologies (**d**)

Only 24 downregulated DEGs were recognized in the osteosarcoma patients that developed metastases, and no upregulated genes were found in the profiles (Fig. 1a), meaning that the DEGs protect patients from metastases. Detailed information for the DEGs is shown in Table 1. The co-expressed DEGs in humans are shown in Fig. 1b. The PPI network of the DEGs is shown in Fig. 1c.

**Table 1:** The statistical metrics for the DEGs

Illumina Probe ID	Gene Symbol	logFC	*p*-value	FDR	t-value	Full name
0003120608	AIF1	−1.2155	2.66E-05	0.027581	− 4.60773	Allograft inflammatory factor 1
0006900465	ALOX5	−1.1467	8.39E-05	0.041468	−4.26677	Arachidonate 5-lipoxygenase
0004180411	ALOX5AP	−1.50813	5.14E-07	0.008347	− 5.72697	Arachidonate 5-lipoxygenase activating protein
0000580603	AMICA1	−1.11325	8.96E-05	0.041468	−4.24687	Junction adhesion molecule like
0004010301	C1orf162	−1.00284	0.000123	0.047543	−4.15122	Chromosome 1 open reading frame 162
0004390370	C1QA	−1.29621	8.78E-05	0.041468	−4.25276	Complement C1q A chain
0000990398	CD14	−1.31053	4.20E-05	0.034058	−4.47365	CD14 molecule
0003420154	CD74	−1.49337	2.02E-05	0.027581	−4.68835	CD74 molecule
0000010215	CD86	−1.14346	5.97E-06	0.01725	−5.03952	CD86 molecule
0002100100	FCGR2A	−1.12434	2.10E-06	0.017075	−5.33441	Fc fragment of igg receptor iia
0005820008	FGL2	−1.22264	3.46E-06	0.01725	−5.19438	Fibrinogen like 2
0007650441	FHL2	−1.0394	8.64E-05	0.041468	−4.2578	Four and a half LIM domains 2
0004150593	FPRL2	−1.09745	1.19E-05	0.021461	−4.84176	Formyl peptide receptor 3
0005080193	GIMAP4	−1.0098	7.16E-05	0.040544	−4.31433	Gtpase, IMAP family member 4
0007200180	HCLS1	−1.06292	5.44E-05	0.036314	−4.39632	Hematopoietic cell-specific Lyn substrate 1
0007400685	HCST	−1.19629	5.03E-05	0.035478	−4.42004	Hematopoietic cell signal transducer
0007400136	HLA-DMA	−1.31531	6.25E-06	0.01725	−5.02628	Major histocompatibility complex, class II, DM alpha
0005870743	HLA-DMB	−1.23415	5.05E-06	0.01725	−5.0869	Major histocompatibility complex, class II, DM beta
0006560088	HLA-DOA	−1.08698	4.89E-06	0.01725	−5.09634	Major histocompatibility complex, class II, DO alpha
0006480500	HLA-DPA1	−1.4719	2.18E-05	0.027581	−4.66677	Major histocompatibility complex, class II, DP alpha 1
0006290561	HLA-DQA1	−1.60434	4.70E-05	0.034712	−4.44	Major histocompatibility complex, class II, DQ alpha 1
0001440296	HLA-DQB1	−1.32328	0.000141	0.04985	−4.10794	Major histocompatibility complex, class II, DQ beta 1
0002680370	HLA-DRA	−1.48612	1.52E-05	0.024614	−4.77169	Major histocompatibility complex, class II, DR alpha
0006040379	HLA-DRB4	−1.59588	3.18E-05	0.03033	−4.55585	Major histocompatibility complex, class II, DR beta 4

### GO and pathway enrichment

**Fig. 2:**
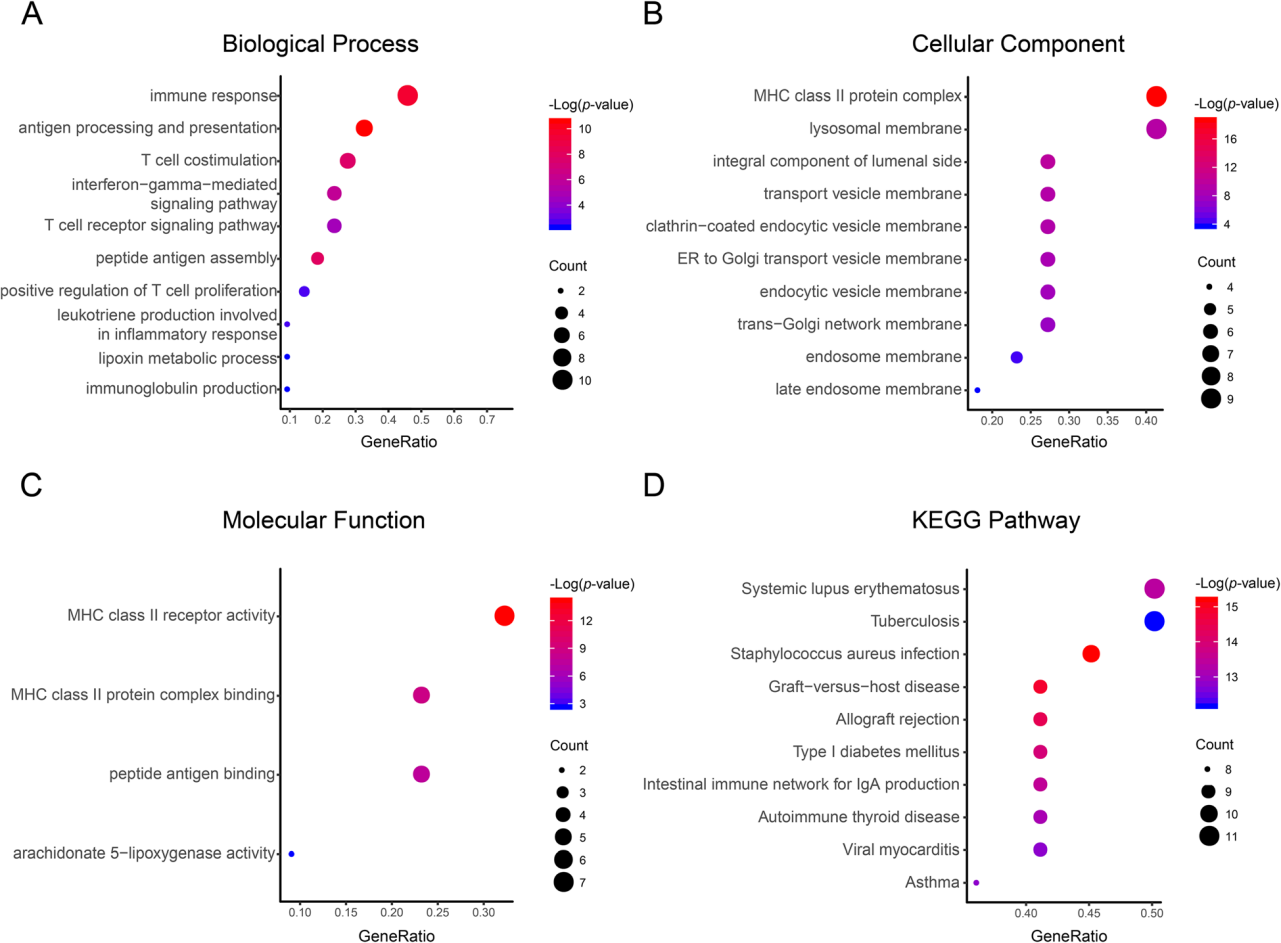
GO and KEGG pathway enrichment analyses of the DEGs including biological process (**a**), cellular component (**b**), and molecular function (**c**). Functional and pathway enrichment analyses were performed using DAVID (**d**). The size of the dots represents the gene count and the color depth of the dots represents the -log (*p*-value)

The results of the biological classification of the DEGs, and functional and pathway enrichment analyses are shown in Fig. 2 (details are shown in Tables 2 and 3). The results of the biological process analysis of the DEGs is shown in Fig. 1d. GO analysis showed that in the BP ontology (Fig. 2a), immune response (10 genes) and T cell co-stimulation (6 genes) constituted the most significantly enriched terms. In the CC ontology (Fig. 2b), the most significantly enriched terms were involved in MHC class II protein complex (9 genes) and the lysosomal membrane (9 genes). In the MF ontology (Fig. 2c), the most significantly enriched terms were involved in MHC class II receptor activity (7 genes), MHC class II protein complex binding (5 genes), and peptide antigen binding (5 genes). In the KEGG pathways (Fig. 2d), the most significantly enriched terms were shown as tuberculosis (11 genes) and systemic lupus erythematosus (11 genes).

**Table 2:** The top 5 enriched GO terms of the DEGs

Category	GO ID	GO Term	Count	FDR	Log P	Genes
Biological Process	0002504	antigen processing and presentation of peptide or polysaccharide antigen via MHC class II	8	1.62E-14	−16.8846	HLA-DQB1, HLA-DRB4, HLA-DPA1, HLA-DMB, HLA-DOA, HLA-DMA, HLA-DQA1, HLA-DRA
Biological Process	0019886	antigen processing and presentation of exogenous peptide antigen via MHC class II	9	5.29E-11	−13.369	HLA-DQB1, HLA-DRB4, HLA-DPA1, HLA-DMB, HLA-DOA, HLA-DMA, CD74, HLA-DQA1, HLA-DRA
Biological Process	0019882	antigen processing and presentation	7	3.03E-08	−10.6113	HLA-DQB1, HLA-DRB4, HLA-DPA1, HLA-DMB, CD74, HLA-DQA1, HLA-DRA
Biological Process	0006955	immune response	10	3.29E-07	−9.57522	HLA-DQB1, CD86, HLA-DRB4, HLA-DPA1, HLA-DMB, HLA-DOA, HLA-DMA, CD74, HLA-DQA1, HLA-DRA
Biological Process	0002503	peptide antigen assembly with MHC class II protein complex	4	1.52E-05	−7.91138	HLA-DRB4, HLA-DMB, HLA-DMA, HLA-DRA
Cellular Component	0042613	MHC class II protein complex	9	2.13E-16	−18.6699	HLA-DQB1, HLA-DRB4, HLA-DPA1, HLA-DMB, HLA-DOA, HLA-DMA, CD74, HLA-DQA1, HLA-DRA
Cellular Component	0071556	integral component of lumenal side of endoplasmic reticulum membrane	6	1.41E-07	−9.84824	HLA-DQB1, HLA-DRB4, HLA-DPA1, CD74, HLA-DQA1, HLA-DRA
Cellular Component	0005765	lysosomal membrane	9	4.03E-07	−9.39244	HLA-DQB1, HLA-DRB4, HLA-DPA1, HLA-DMB, HLA-DOA, HLA-DMA, CD74, HLA-DQA1, HLA-DRA
Cellular Component	0030658	transport vesicle membrane	6	5.93E-07	−9.2251	HLA-DQB1, HLA-DRB4, HLA-DPA1, CD74, HLA-DQA1, HLA-DRA
Cellular Component	0030669	clathrin-coated endocytic vesicle membrane	6	8.83E-07	−9.05199	HLA-DQB1, HLA-DRB4, HLA-DPA1, CD74, HLA-DQA1, HLA-DRA
Molecular Function	0032395	MHC class II receptor activity	7	5.72E-12	−14.2221	HLA-DQB1, HLA-DRB4, HLA-DPA1, HLA-DOA, HLA-DMA, HLA-DQA1, HLA-DRA
Molecular Function	0023026	MHC class II protein complex binding	5	2.47E-06	−8.58782	HLA-DMB, HLA-DOA, HLA-DMA, CD74, HLA-DRA
Molecular Function	0042605	peptide antigen binding	5	2.75E-05	−7.54062	HLA-DQB1, HLA-DRB4, HLA-DPA1, HLA-DQA1, HLA-DRA
Molecular Function	0004051	arachidonate 5-lipoxygenase activity	2	2.237376	−2.62558	ALOX5AP, ALOX5

**Table 3:** The enriched KEGG pathway terms of the DEGs

Pathway ID	Pathway Name	Gene Count	FDR	LogP	Genes
hsa05150	*Staphylococcus aureus* infection	10	6.33E-13	−15.21	HLA-DQB1, C1QA, HLA-DRB4, HLA-DPA1, FCGR2A, HLA-DMB, HLA-DOA, HLA-DMA, HLA-DQA1, HLA-DRA
hsa05332	Graft-versus-host disease	9	1.37E-12	−14.85	HLA-DQB1, CD86, HLA-DRB4, HLA-DPA1, HLA-DMB, HLA-DOA, HLA-DMA, HLA-DQA1, HLA-DRA
hsa05330	Allograft rejection	9	3.67E-12	−14.41	HLA-DQB1, CD86, HLA-DRB4, HLA-DPA1, HLA-DMB, HLA-DOA, HLA-DMA, HLA-DQA1, HLA-DRA
hsa04940	Type I diabetes mellitus	9	1.11E-11	−13.93	HLA-DQB1, CD86, HLA-DRB4, HLA-DPA1, HLA-DMB, HLA-DOA, HLA-DMA, HLA-DQA1, HLA-DRA
hsa04672	Intestinal immune network for IgA production	9	2.96E-11	−13.50	HLA-DQB1, CD86, HLA-DRB4, HLA-DPA1, HLA-DMB, HLA-DOA, HLA-DMA, HLA-DQA1, HLA-DRA
hsa05322	Systemic lupus erythematosus	11	3.85E-11	−13.39	HLA-DQB1, C1QA, CD86, HLA-DRB4, HLA-DPA1, FCGR2A, HLA-DMB, HLA-DOA, HLA-DMA, HLA-DQA1, HLA-DRA
hsa05320	Autoimmune thyroid disease	9	7.06E-11	−13.13	HLA-DQB1, CD86, HLA-DRB4, HLA-DPA1, HLA-DMB, HLA-DOA, HLA-DMA, HLA-DQA1, HLA-DRA
hsa05310	Asthma	8	1.49E-10	−12.80	HLA-DQB1, HLA-DRB4, HLA-DPA1, HLA-DMB, HLA-DOA, HLA-DMA, HLA-DQA1, HLA-DRA
hsa05416	Viral myocarditis	9	1.54E-10	−12.79	HLA-DQB1, CD86, HLA-DRB4, HLA-DPA1, HLA-DMB, HLA-DOA, HLA-DMA, HLA-DQA1, HLA-DRA
hsa05152	Tuberculosis	11	6.54E-10	−12.16	HLA-DQB1, HLA-DRB4, HLA-DPA1, FCGR2A, HLA-DMB, HLA-DOA, HLA-DMA, CD14, CD74, HLA-DQA1, HLA-DRA
hsa05140	Leishmaniasis	9	9.79E-10	−11.98	HLA-DQB1, HLA-DRB4, HLA-DPA1, FCGR2A, HLA-DMB, HLA-DOA, HLA-DMA, HLA-DQA1, HLA-DRA
hsa04612	Antigen processing and presentation	9	1.73E-09	−11.74	HLA-DQB1, HLA-DRB4, HLA-DPA1, HLA-DMB, HLA-DOA, HLA-DMA, CD74, HLA-DQA1, HLA-DRA
hsa05323	Rheumatoid arthritis	9	5.81E-09	−11.21	HLA-DQB1, CD86, HLA-DRB4, HLA-DPA1, HLA-DMB, HLA-DOA, HLA-DMA, HLA-DQA1, HLA-DRA
hsa04145	Phagosome	10	8.33E-09	−11.06	HLA-DQB1, HLA-DRB4, HLA-DPA1, FCGR2A, HLA-DMB, HLA-DOA, HLA-DMA, CD14, HLA-DQA1, HLA-DRA
hsa05145	Toxoplasmosis	9	3.62E-08	−10.42	HLA-DQB1, HLA-DRB4, HLA-DPA1, ALOX5, HLA-DMB, HLA-DOA, HLA-DMA, HLA-DQA1, HLA-DRA
hsa05321	Inflammatory bowel disease (IBD)	8	4.36E-08	−10.34	HLA-DQB1, HLA-DRB4, HLA-DPA1, HLA-DMB, HLA-DOA, HLA-DMA, HLA-DQA1, HLA-DRA
hsa04514	Cell adhesion molecules (CAMs)	9	2.86E-07	−9.52	HLA-DQB1, CD86, HLA-DRB4, HLA-DPA1, HLA-DMB, HLA-DOA, HLA-DMA, HLA-DQA1, HLA-DRA
hsa05168	Herpes simplex infection	9	2.18E-06	−8.64	HLA-DQB1, HLA-DRB4, HLA-DPA1, HLA-DMB, HLA-DOA, HLA-DMA, CD74, HLA-DQA1, HLA-DRA
hsa05164	Influenza A	8	5.24E-05	−7.26	HLA-DQB1, HLA-DRB4, HLA-DPA1, HLA-DMB, HLA-DOA, HLA-DMA, HLA-DQA1, HLA-DRA
hsa05166	HTLV-I infection	8	7.01E-04	−6.13	HLA-DQB1, HLA-DRB4, HLA-DPA1, HLA-DMB, HLA-DOA, HLA-DMA, HLA-DQA1, HLA-DRA
hsa05169	Epstein-Barr virus infection	5	0.13728	−3.84	HLA-DQB1, HLA-DRB4, HLA-DPA1, HLA-DQA1, HLA-DRA
hsa04640	Hematopoietic cell lineage	3	14.8897	−1.77	HLA-DRB4, CD14, HLA-DRA

### Survival analysis of the DEGs

**Fig. 3:**
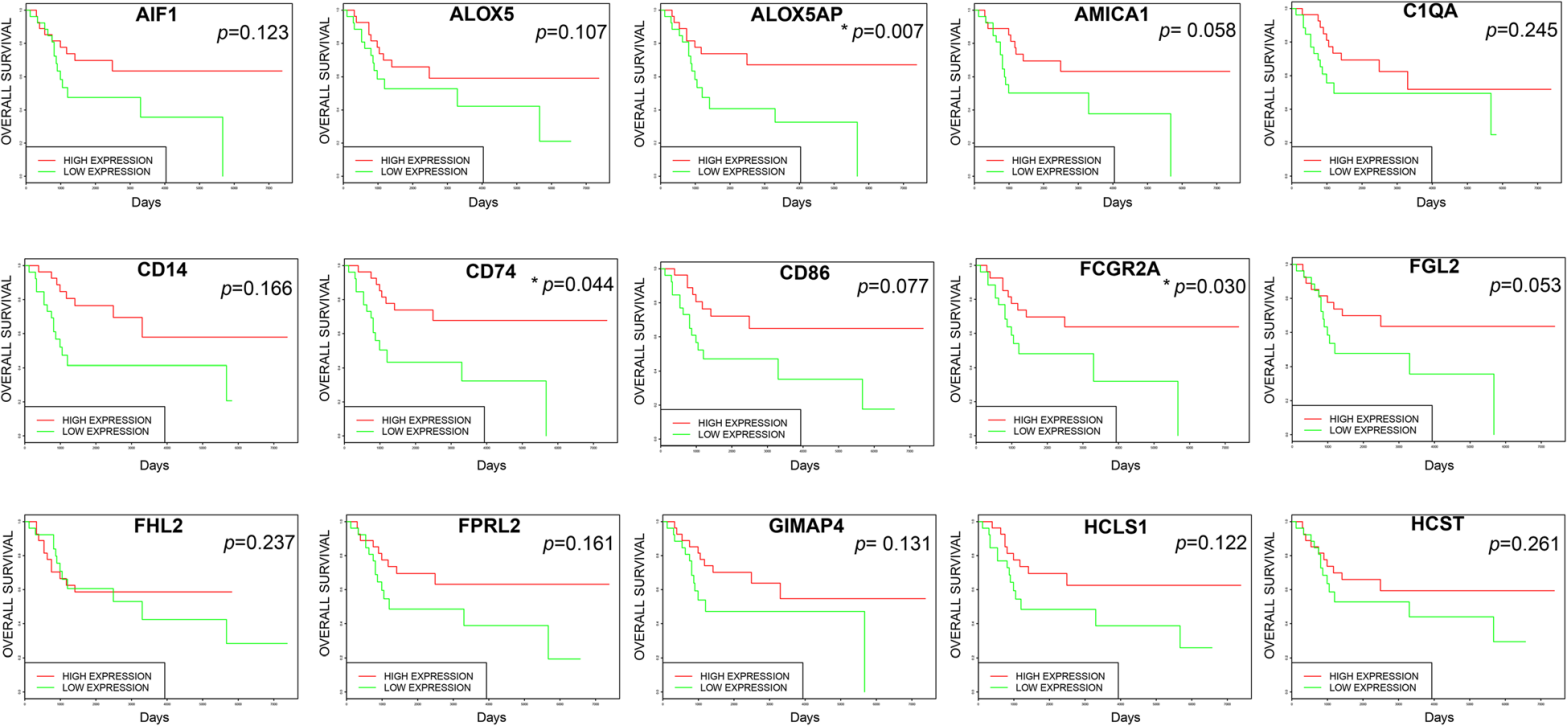
Survival curves of DEGs were created using the Kaplan-Meier curve in the PROGgeneV2 online platform; the red line represents the high expression of the gene and the green line represents the low expression of the gene

Among the 24 DEGs, overall survival plots were obtained for 15 genes, as shown in Fig. 3. The high expression group of 15 DEGs would have better survival than the low expression group. However, only three of these were significant (< 0.05), namely ALOX5AP, CD74, and FCGR2A. These were selected as the candidate genes for further analyses. The gene expression of the candidate genes could be found in the Additional file 1: Table S1.

### Function predictions for the candidate genes

**Fig. 4:**
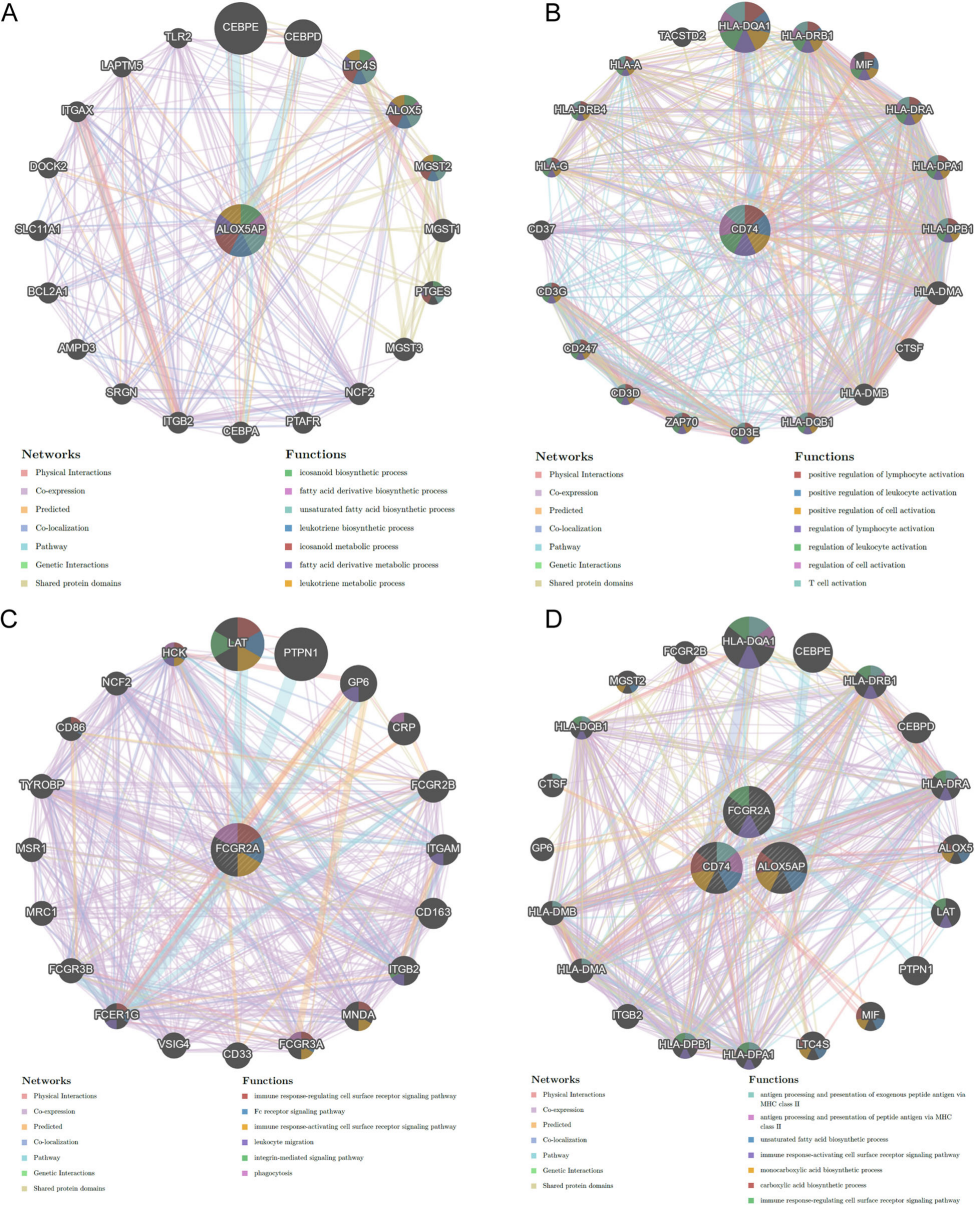
Protein-protein interaction network of ALOX5AP (**a**), CD74 (**b**), and FCGR2A (**c**) candidate genes (**d**). Different colors of the network edges indicate the bioinformatics method applied; the different colors for the network nodes indicate the biological functions of the set of enrichment genes

An interactive functional association network constructed by GeneMANIA revealed correlations among genes for the candidate genes. The gene set enriched for ALOX5AP is responsible mainly for eicosanoid and fatty acid derivative biosynthetic processes (Fig. 4a). Meanwhile, the gene set enriched for CD74 is responsible mainly for positive regulation of lymphocyte activation and leukocyte activation (Fig. 4b), and the gene set enriched for FCGR2A is responsible for immune response-regulating cell surface receptor signaling pathways, and Fc receptor signaling pathways (Fig. 4c). Moreover, the gene set enriched for the three genes is responsible mainly for antigen processing and presentation of exogenous peptide antigens via MHC class II, antigen processing, and presentation of peptide antigens via MHC class II (Fig. 4d). Compared to the functional analyses of the DEGs, the enriched functions of the candidate genes also have their own characteristics.

### Different expression of candidate genes in normal and malignant human tissues

**Fig. 5:**
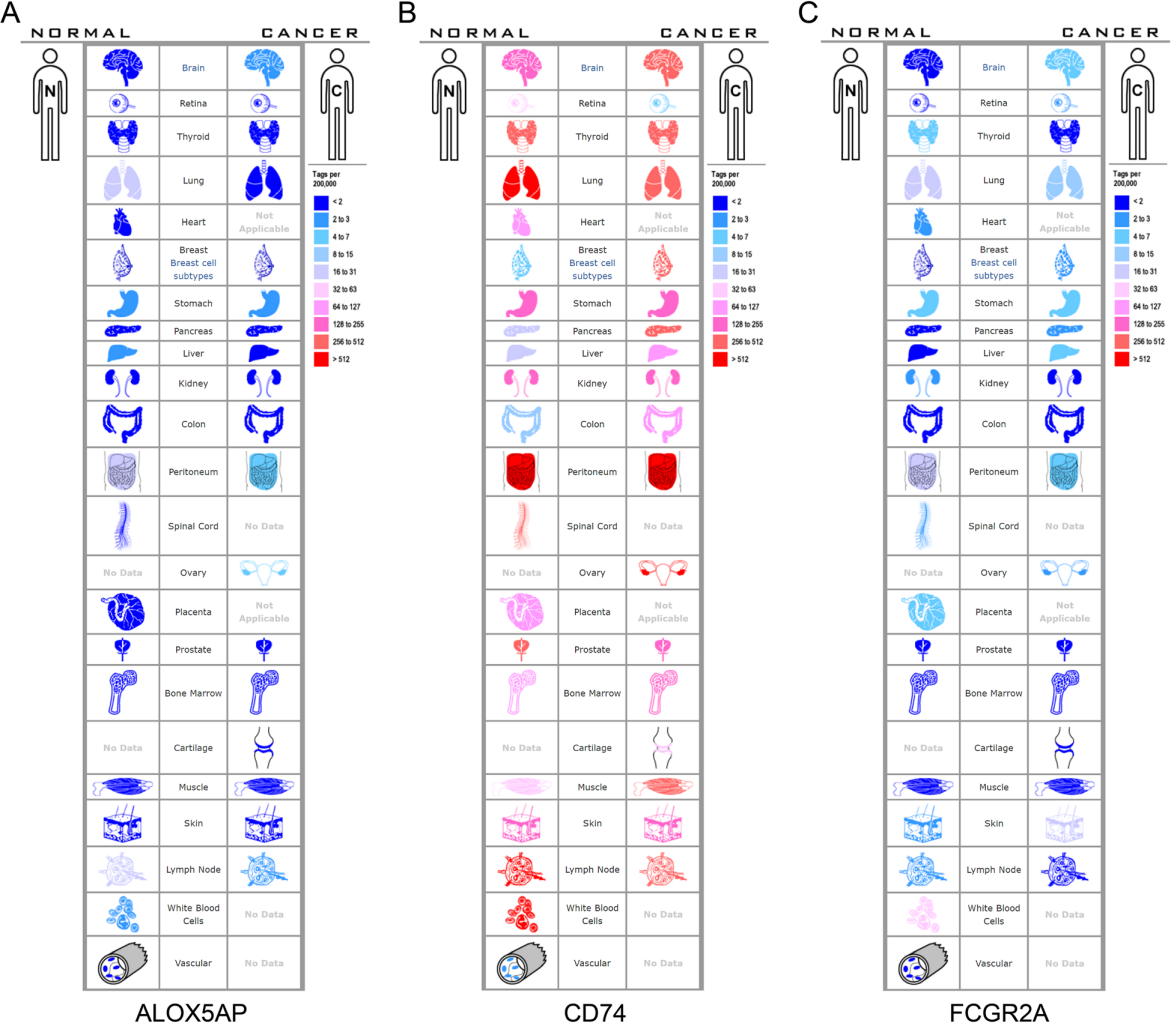
Expression profiles for ALOX5AP (**a**), CD74 (**b**), and FCGR2A (**c**) in human cancers analyzed using SAGE. The left side represents normal tissues and the right side represents the matched cancer tissues. The related expression levels are based on the analysis of counts of SAGE tags, ordered by ten colors

The expression profiles of the three candidate genes in human tissue were displayed using SAGE. As shown, ALOX5AP mRNA in lung, liver, breast, peritoneum, and lymph node tissues displayed higher levels than in the matched cancer tissues (Fig. 5a). CD74 mRNA in brain, retina, lung, and lymph nodes displayed higher levels than in the matched cancer tissues (Fig. 5b), while FCGR2A mRNA in thyroid, lung, kidney, peritoneum, and lymph node tissues displayed higher levels than in the matched cancer tissues (Fig. 5c). All the candidate genes were expressed at higher levels in lung and lymph node tissues than in the matched cancer tissues.

### Immune infiltration analysis of the candidate genes

**Fig. 6:**
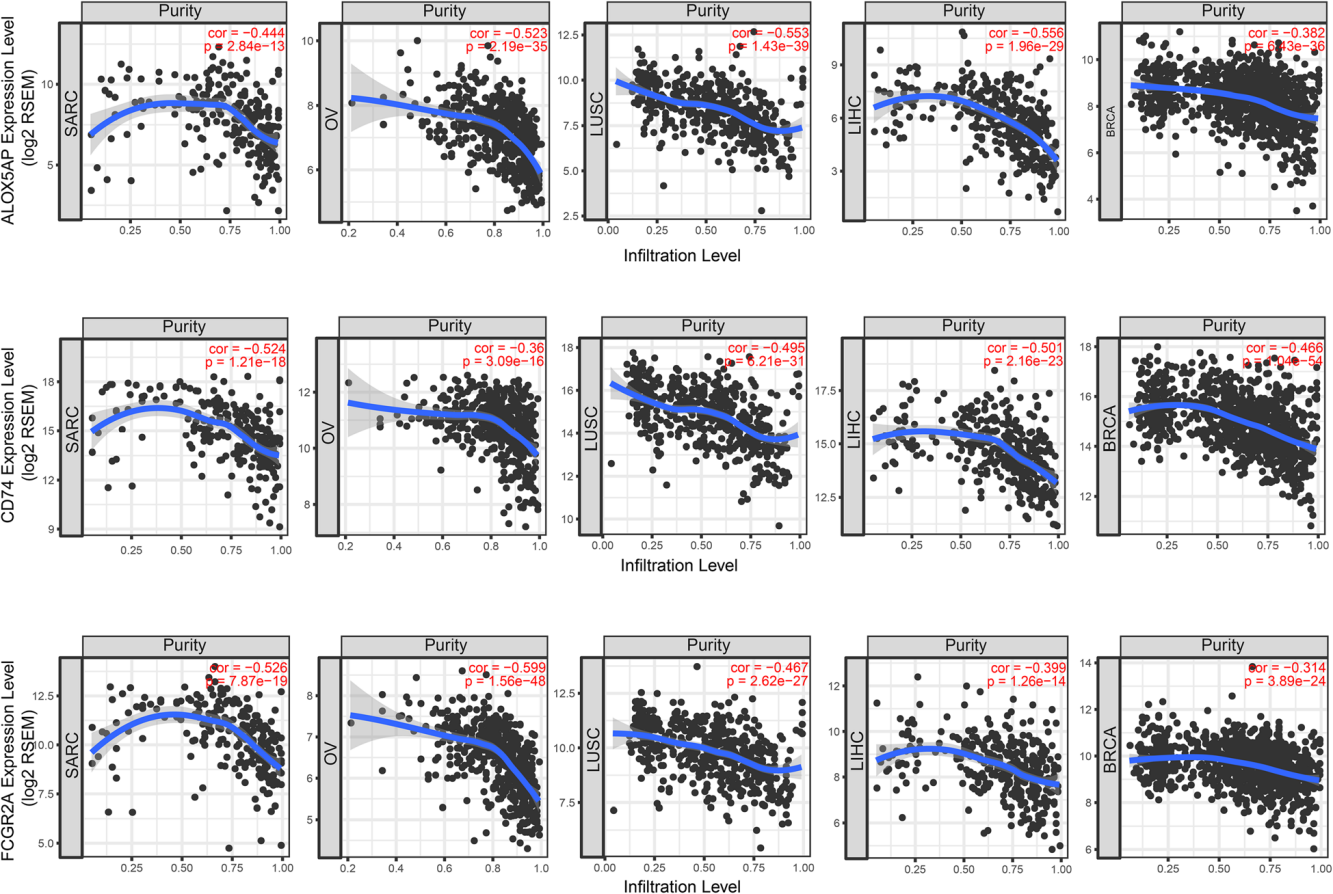
Immune infiltration of ALOX5AP (A), CD74 (B), and FCGR2A (C) in different cancer types, such as SARC (sarcoma), OV (ovarian serous cystadenocarcinoma), LUSC (lung squamous cell carcinoma), LIHC (liver hepatocellular carcinoma), and BRCA (breast invasive carcinoma)

In the five cancer types we selected, the expression levels of the three candidate genes were all negatively associated with tumor purity (Fig. 6). It can be inferred from this result that all three candidate genes are probably expressed in the microenvironment, not in the tumor cells.

The data that support the findings of this study were generated at GSE21257 [12] in GEO. Derived data supporting the findings of this study are available from the corresponding author on request.

## Discussion

Osteosarcoma metastasis is a complex process of interaction between multiple genes and multiple signaling pathways in tumor cells and stromal cells. The deletion of the p53 gene and activation of the Notch pathway in osteosarcoma cells may contribute to invasion and metastasis [24]. Induction of Src-family tyrosine kinase (SFK) and the synergy of metal matrix protease-2, 9 (MMPs-2, 9), may help osteosarcoma cells degrade the extracellular matrix and enter the blood circulation by activating the Wnt/beta-catenin signaling pathway [25]. Meanwhile, SFK activates PI3K/AKT and Ras/MAPK signaling pathways to avoid apoptosis in osteosarcoma cells [26].

Buddingh et al. first reported the series in 2011 [12] which we analyzed in this study. They also compared patients that did, or did not, develop metastases within 5 years and identified 14 upregulated and 118 downregulated genes in patients that developed metastases, with an only statistical criterion of adjust *p* < 0.05. Buddingh proved that these genes were expressed by tumor stroma and not by tumor cells by experiment. Almost half of these genes were attributed to macrophage function. Furthermore, the authors proposed that tumor-associated macrophages (TAM) in the tumor-microenvironment have an antimetastatic effect, which can improve survival in osteosarcoma.

This is a notable work. However, the considered statistical criteria was just only the *p*-value and may produce some false-positive results. Meanwhile, the authors focused on the antimetastatic function of TAM and provided a detailed argument to support this. They did not identify the key molecules played a role in this process which would be benefit for future researchers. In our study, only 24 downregulated DEGs were recognized with a statistical significance of adjust *p* < 0.05 and absolute value of fold change > 2. These DEGs have greater statistical significance than those in the previous study. Besides that, we proposed three prognostic candidate genes would play an important role in the patients who did develop metastases within 5 years.

GO and KEGG pathway enrichment analyses revealed that changes in the DEGs mainly occurred in the MHC class II protein complex, immune response, and antigen processing and presentation. In other words, immune infiltrates or immune responses in the local microenvironment play an important role in osteosarcoma metastasis. Previous studies have reported that during metastasis, tumor-infiltrating lymphocytes (TILs) can be detected at a higher level than in normal tissue [27], and patients with higher T-lymphocyte infiltration showed improved survival [28, 29]. It was proposed that some portion of the T-cells (like TILs) would act against tumor cells with a higher specific immunological reactivity than the non-infiltrating lymphocytes [27]. Moreover, programmed cell death protein 1(PD-1) showed increased expressed in TIL [30] and peripheral CD4^+^ and CD8^+^ T-lymphocytes [31] Based on this result, the inhibition of the PD-1/PDL-1 interaction would lead to a decreased tumor burden in osteosarcoma-bearing mice [32]. Overall, these theories are in agreement with our results.

Three candidate genes with prognostic value—namely ALOX5AP, CD74, and FCGR2A—were discovered. Interestingly, all the candidate genes showed higher expression in lung and lymph node tissues than in the matched cancer tissues and were probably expressed in the microenvironment, not in the tumor cells. This result is consistent with that of previous studies; the candidate genes are reportedly linked to tumor cells. The change in ALOX5AP expression can cause oxidative stress, which has some effects on human leukemia [33]. Codreanu et al. reported that ALOX5AP could be a noninvasive candidate biomarker for lung cancer with global and targeted proteomics [34]. Knights et al. identified ALOX5AP as associated with the pharmacokinetics of gemcitabine, which is an approved anti-cancer drug [35]. Meanwhile, high expression of CD74 would cause functional HLA class II processing in brain metastatic tumor cells, with a better prognosis [36]. Figueiredo et al. reported that MIF-CD74 signaling regulates the antitumor immune response of macrophages and dendritic cells in metastatic melanoma [37]. Ekmekcioglu et al. found that CD74 is associated with overall survival and recurrence-free survival in stage III melanoma, and could be a useful prognostic tumor marker [38]. Furthermore, FCGR2A is reportedly associated with the pharmacodynamics of monoclonal antibodies in different cancer types, such as colorectal cancer [39], breast cancer [40], and metastatic squamous cell head and neck cancer [41]. However, a search of the published literature revealed that there are few studies about the candidate genes for osteosarcoma. This observation suggests that the candidate genes may require further research to reveal the mechanisms of osteosarcoma metastasis.

This bioinformatics study provides information on the DEGs and candidate genes that protect osteosarcoma patients from metastasis, which could inform future research. However, we must recognize that the roles of the candidate genes are still unknown. Additional well-designed experiments and analyses are required to reveal these mechanisms. In addition, all the results from this study were obtained in silicon; in vivo and in vitro experiments are necessary to test the functions of these DEGs. A note that if we could include some critical details of the surrounding muscle tissue, we might better analyze the mechanism of osteosarcoma metastasis.

## Conclusions

In conclusion, we identified 24 DEGs, of which three candidate genes may be involved in the processes that protect osteosarcoma patients from metastasis. The molecules we found are potential targets for future research on osteosarcoma immunity. Furthermore, our results contribute to the identified biomarkers for osteosarcoma metastasis.

## Supplementary information

**Additional file 1: Table S1.** The gene expression of the candidate genes.

## Data Availability

The data that support the findings of this study were generated at GSE21257(Buddingh et al., 2011) in GEO. Derived data supporting the findings of this study are available from the corresponding author on reasonable request.

## Abbreviations

DEGs: Differentially expressed genes
GEO: Gene Expression Omnibus
GO: Gene Ontology
KEGG: Kyoto Encyclopedia of Genes and Genomes
PPI: Protein-protein interaction
SAGE: Serial Analysis of Gene Expression
STRING: Search Tool for the Retrieval of Interacting Genes
TIMER: Tumor IMmune Estimation Resource
