# Supplementary material for: Key genes with prognostic values in suppression of osteosarcoma metastasis using comprehensive analysis
Source: BMC Cancer. 2020 Jan 28;20:65. doi: 10.1186/s12885-020-6542-z (PMC6988291; doi:10.1186/s12885-020-6542-z)
Supplement: Supplementary file 1 — Additional file 1: Table S1. The gene expression of the candidate genes. [file 12885_2020_6542_MOESM1_ESM.docx]

Table S1 The gene expression of the candidate genes

| GEO- ID | ALOX5AP | CD74 | FCGR2A |
| --- | --- | --- | --- |
| GSM530667 | 10.19798 | 9.300785 | 7.667956 |
| GSM530899 | 9.684193 | 8.437351 | 7.873772 |
| GSM531283 | 9.229609 | 7.428951 | 7.114627 |
| GSM531284 | 10.32094 | 8.86508 | 7.479745 |
| GSM531285 | 9.716864 | 8.337736 | 7.820033 |
| GSM531286 | 9.529429 | 9.611414 | 7.659545 |
| GSM531287 | 11.44733 | 11.55449 | 8.650575 |
| GSM531288 | 11.21959 | 11.07535 | 9.219106 |
| GSM531289 | 10.49737 | 8.131829 | 7.54691 |
| GSM531290 | 8.608282 | 8.151233 | 7.435858 |
| GSM531291 | 9.885699 | 8.932875 | 7.644844 |
| GSM531292 | 10.20786 | 10.26089 | 8.703376 |
| GSM531293 | 10.38484 | 10.89083 | 7.902349 |
| GSM531294 | 9.339081 | 8.284635 | 7.688458 |
| GSM531295 | 9.646966 | 8.460747 | 7.460232 |
| GSM531296 | 10.03453 | 9.232795 | 7.66034 |
| GSM531297 | 11.28602 | 8.988639 | 7.916053 |
| GSM531298 | 8.600827 | 7.749987 | 7.128128 |
| GSM531299 | 9.916586 | 8.543269 | 8.156171 |
| GSM531300 | 10.64638 | 8.847851 | 7.848885 |
| GSM531301 | 10.84248 | 9.154844 | 8.143059 |
| GSM531302 | 12.42726 | 10.74824 | 8.160256 |
| GSM531303 | 12.89011 | 11.65507 | 9.374091 |
| GSM531304 | 12.45172 | 10.39846 | 9.616074 |
| GSM531305 | 12.94877 | 10.87044 | 9.334836 |
| GSM531306 | 10.30278 | 10.11893 | 8.925138 |
| GSM531307 | 12.24036 | 11.94748 | 9.642116 |
| GSM531308 | 11.02336 | 8.779001 | 8.465923 |
| GSM531309 | 11.89919 | 11.90642 | 9.303225 |
| GSM531310 | 11.57055 | 11.11676 | 9.462329 |
| GSM531311 | 11.2605 | 9.44014 | 8.669066 |
| GSM531312 | 10.95225 | 8.636531 | 7.895033 |
| GSM531313 | 10.69317 | 11.02275 | 9.775824 |
| GSM531314 | 11.16997 | 9.667501 | 8.580173 |
| GSM531319 | 11.01975 | 10.58378 | 8.563786 |
| GSM531320 | 12.80555 | 11.54473 | 9.620978 |
| GSM531321 | 12.61282 | 11.93523 | 11.0741 |
| GSM531322 | 11.58797 | 11.21866 | 8.996408 |
| GSM531323 | 12.24194 | 11.66468 | 10.15595 |
| GSM531324 | 10.79417 | 11.72478 | 8.88706 |
| GSM531325 | 10.89392 | 9.902986 | 8.646289 |
| GSM531326 | 11.85737 | 10.51363 | 9.078614 |
| GSM531327 | 8.766162 | 8.255822 | 7.67679 |
| GSM531328 | 12.01965 | 10.02195 | 9.035205 |
| GSM531329 | 9.47744 | 7.288835 | 7.152013 |
| GSM531330 | 12.28952 | 10.94464 | 10.09306 |
| GSM531331 | 11.28106 | 10.50332 | 8.71719 |
| GSM531332 | 11.74063 | 10.5327 | 9.20831 |
| GSM531333 | 12.02814 | 11.67185 | 9.651106 |
| GSM531334 | 11.36984 | 10.34391 | 8.690044 |
| GSM531335 | 13.21488 | 11.65656 | 10.21661 |
| GSM531351 | 11.83177 | 10.35908 | 8.989561 |
| GSM531352 | 11.17218 | 10.28088 | 8.402515 |
